# The population genetics of cooperative gene regulation

**DOI:** 10.1186/1471-2148-12-173

**Published:** 2012-09-06

**Authors:** Alexander J Stewart, Robert M Seymour, Andrew Pomiankowski, Joshua B Plotkin

**Affiliations:** 1 Department of Biology, University of Pennsylvania, Philadelphia, PA, USA; 2 CoMPLEX, University College London, London, UK; 3 Department of Mathematics, University College London, London, UK; 4 Department of Genetics, Evolution and Environment, University College London, London, UK

## Abstract

**Background:**

Changes in gene regulatory networks drive the evolution of phenotypic diversity both within and between species. Rewiring of transcriptional networks is achieved either by changes to transcription factor binding sites or by changes to the physical interactions among transcription factor proteins. It has been suggested that the evolution of cooperative binding among factors can facilitate the adaptive rewiring of a regulatory network.

**Results:**

We use a population-genetic model to explore when cooperative binding of transcription factors is favored by evolution, and what effects cooperativity then has on the adaptive re-writing of regulatory networks. We consider a pair of transcription factors that regulate multiple targets and overlap in the sets of target genes they regulate. We show that, under stabilising selection, cooperative binding between the transcription factors is favoured provided the amount of overlap between their target genes exceeds a threshold. The value of this threshold depends on several population-genetic factors: strength of selection on binding sites, cost of pleiotropy associated with protein-protein interactions, rates of mutation and population size. Once it is established, we find that cooperative binding of transcription factors significantly accelerates the adaptive rewiring of transcriptional networks under positive selection. We compare our qualitative predictions to systematic data on *Saccharomyces cerevisiae* transcription factors, their binding sites, and their protein-protein interactions.

**Conclusions:**

Our study reveals a rich set of evolutionary dynamics driven by a tradeoff between the beneficial effects of cooperative binding at targets shared by a pair of factors, and the detrimental effects of cooperative binding for non-shared targets. We find that cooperative regulation will evolve when transcription factors share a sufficient proportion of their target genes. These findings help to explain empirical pattens in datasets of transcription factors in *Saccharomyces cerevisiae* and, they suggest that changes to physical interactions between transcription factors can play a critical role in the evolution of gene regulatory networks.

## Background

It is often difficult for a population to acquire an adaptive phenotype that requires simultaneous changes in the co-expression of multiple genes [1-10]. If selection favours a change in the way a group of genes are regulated, then each of the target genes must independently gain novel binding sites and/or lose existing ones [8,9,11,12]. This has led to the proposal that adaptive rewiring of a regulatory network can be accelerated if pairs of transcription factors bind their targets cooperatively, through a physical interaction between the transcription factor proteins themselves [8,9,11].

**Figure 1:**
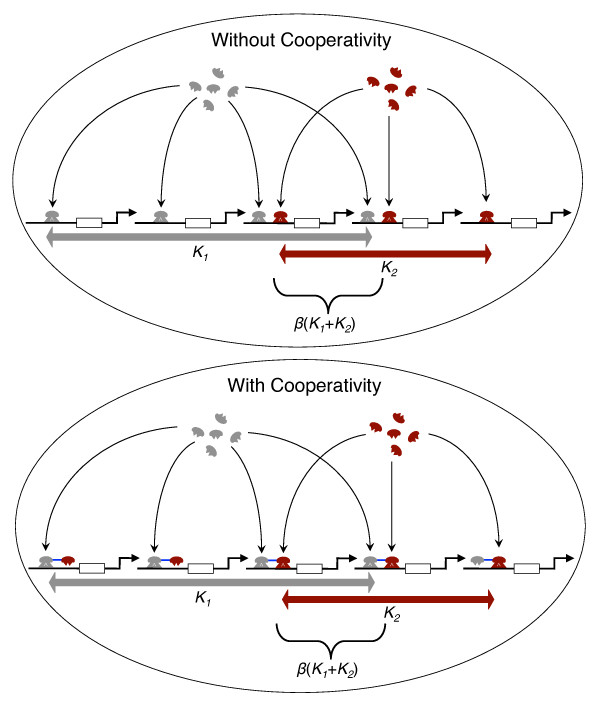
**Schematic of the population-genetic model.** A schematic cartoon of our population-genetic model. (top) When cooperativity is absent different transcription factors (gray and red) must bind to sites at each of their targets independently. Each factor has a number of targets, *K*_1_and *K*_2_, and a number *β*(*K*1 + *K*2) of shared targets (bottom). When cooperativity is present, a physical interaction between transcription factors (blue line) can mitigate the need to bind independently at shared targets, but may cause misregulation at targets that are not shared, by causing the factor with which it interacts cooperatievly to misbind. Cooperatively is therefore advantageous between transcription factors that share many targets, but it may be deleterious at targets that are not shared.

Given the potential adaptive benefit of cooperative regulation, it makes sense to ask, when will cooperative binding between a pair of transcription factors be able to invade a population that lacks such cooperativity? To answer this we must understand the following tradeoff: although cooperative binding between a pair of factors may result in improved regulation at the target genes shared by both factors, any mutation that results in a physical interaction between the transcription factors will effect *all of their targets* (Figure 1). Thus the advantageous fitness effects of improved binding at some, shared targets must outweigh any deleterious effects of misregulation at other, non-shared targets in order for cooperative binding to be favored by evolution.

A number of previous studies have explored the mechanistic details of cooperative transcription factor binding at a given target gene [13-15]. Such biophysical studies focus on transcription factor binding at a single target gene and are able, with remarkable accuracy, to account for a number of the physical properties of binding sites [14,16,17]. However, the evolution of cooperative binding occurs through mutations at transcription factor proteins, and such mutations can alter transcription factor binding at every binding site across the genome. To understand the fitness effects of such a mutation therefore requires that we understand the evolution of the whole ensemble of binding sites for a transcription factor. The population genetics of such an ensemble cannot be understood in a simple way just by focusing on the details of a single member of the ensemble. They depend critically on the population-genetic parameters of the ensemble, such as number of target genes, overall mutation rates and selection coefficients, and population size. Therefore in this paper, we do not focus on the details of a cooperative binding at a single target gene. Instead our analysis is in terms of these population-genetic parameters, and whilst we estimate selective coefficients from biophysical studies, we do not specify the mechanistic details of protein-DNA interactions that give rise to them.

We use a mathematical model to study the conditions under which cooperative binding between pairs of transcription factors is favoured. We first determine the evolutionary conditions that favour cooperative binding under stabilising selection, in terms of the basic evolutionary parameters of the population: the strength of selection on binding sites, the rate of mutation, and the population size. We then study the influence of cooperative regulation on the capacity for a transcriptional circuit to adapt under positive selection. We calculate the time required for a target gene to gain a new, adaptive transcription factor binding site, in the presence or absence of cooperative interactions among its regulators. We confirm our analytical results on the evolution of cooperative regulation by comparison to Monte-Carlo simulations of the Wright-Fisher process associated with our system, and we compare our qualitative conclusions to systematic empirical data.

Our population-genetic model describes a pair of transcription factors, each with its own set of target genes, with some degree of overlap between these sets (Figure 1). According to our model, which is specified in detail below, a target gene that is regulated by both factors has two corresponding binding sites, while a target gene that is regulated by only one of the factors has a single binding site. We assume that mutations that result in loss of function can occur at any binding site, and that non-functional binding sites can also undergo gain of function mutations. When there is no cooperative regulation between the two transcription factors, binding to each of their targets is determined solely by their binding sites. If a binding site is not functional, this results in reduced fitness. When cooperative binding is present, two conflicting effects occur: On the one hand, cooperative binding partially compensates for the deleterious effects of loss of function mutations to the binding sites at shared targets. On the other hand, cooperative binding results in some degree of mis-regulation at each of the targets that are not shared, and this has a deleterious impact on fitness. By constructing our model in terms of these fitness benefits and costs we are able to study the evolutionary dynamics of the system, and determine the effects of varying different population-genetic parameters on the evolution of cooperative gene regulation. This approach therefore complements the detailed mechanistic models of gene regulation that have been studied elsewhere [13-15].

## Results and discussion

### Stabilising selection without cooperative binding

We consider a pair of transcription factors, labelled 1 and 2, that have *K*_1_ and *K*_2_ targets, respectively. A fraction *β* of the binding sites are at shared target genes, so that the number of binding sites at genes that are co-regulated by the pair is *β*(*K*_1_ + *K*_2_), as illustrated in Figure 1. Loss of function mutations occur at binding sites at a rate *u*_*l*_, and back mutations, which result in a functional binding site being gained at a target, occur at rate *u*_*g*_. An individual incurs a fitness penalty *s*, where 0≤*s* < 1, for each non-functional binding site, and fitness is assumed to be multiplicative across loci. Therefore the fitness of an individual that lacks *i*≤*K*_1_ + *K*_2_ of its required binding sites is *w*_*i*_=(1−*s*)^*i*^. The fitness landscape associated with our model thus has a single peak at *i*=0; and for each transcription factor binding site that is lost, fitness is reduced by an additional factor (1−*s*). Empirical estimates of the strength of selection on transcription factor binding sites suggest that typically *Ns*∼10 [18], suggesting that *s* is small. We assume that *s* is the same for all binding sites, an assumption which we relax in the Methods section.

We consider a population of *N* asexual individuals. The evolution of the population can be described by keeping track of the relative abundances of each “hamming class” [19-21]. Hamming class *i* corresponds to those individuals who currently lack *i* transcription factor binding sites. We denote the frequency of individuals in hamming class *i* by *x*_*i*_. In an infinitely large population, the evolution of hamming class *i* is then described by the differential equations [20,21]

(1) $${\dot{x}}_{i}=\overset{K_{1}+K_{2}}{\underset{j=0}{\sum }}\frac{w_{i}}{\bar{w}}z_{i}P_{\mathrm{ij}},$$

where $\bar{w}=\overset{K_{1}+K_{2}}{\underset{i=0}{\sum }}w_{i}x_{i}$, and *P*_*ij*_ is the probability a genotype lacking *j* functional binding sites mutates to a genotype lacking *i* functional binding sites (see Methods). Previous work [19-21] has shown that at equilibrium, when rates of forward and back mutations are identical (*u*_*l*_=*u*_*g*_), the solution to Equation 1 is a binomial distribution. In the more general case of a finite population, with *u*_*l*_≠*u*_*g*_, we find that the equilibrium continues to be well approximated by a binomial distribution, with mean (*K*_1_ + *K*_2_)*a*_*s*_. The term *a*_*s*_ is the probability that a binding site will be non-functional in a randomly chosen individual at equilibrium. The probability *a*_*s*_ depends on the strength of selection against non-functional binding sites, *s*, population size, *N*, and the rates of forward and back mutation, *u*_*l*_and *u*_*g*_ (see Methods and [20,21]).

The equilibrium distribution above describes how stabilizing selection determines the frequencies of functional binding sites in a population. The associated mean fitness for a pair of transcription factors that do not bind cooperatively is $\bar{w}={\left(1-a_{s}s\right)}^{K_{1}+K_{2}}$ (see Methods), and the mean fitness contribution of each binding site is 1−*a*_*s*_*s*. We are typically concerned with the case in which *u*_*l*_,*u*_*g*_ ≪ *s*. In this case, when 2*Ns* > 1, *a*_*s*_ can be approximated by

$$a_{s}\approx \frac{1}{2\mathrm{Ns}}\frac{u_{l}}{u_{l}+u_{g}}+\frac{u_{l}}{s}$$

and otherwise by

(2) $$a_{s}\approx \frac{u_{l}}{u_{l}+u_{g}}$$

(see Methods). These equations have an intuitive interpretation: When 2*Ns* > 1 the first term describes the effect of genetic drift which tends to push the system towards its neutral equilibrium, *a*_0_=*u*_*l*_/(*u*_*l*_ + *u*_*g*_), and the second term describes the effect of selection. In the limit *N*→*∞*, *a*_*s*_ equals *u*_*l*_/*s*, which is the standard result for the frequency of a deleterious allele in an infinite population under mutation-selection balance. When 2*Ns* < 1, evolution is nearly neutral and drift dominates, so the system is close to the neutral equilibrium *a*_0_.

### Stabilising selection with cooperative binding

Here we modify our model to account for cooperative regulation by a pair of factors. This allows us to ask when cooperative regulation is favored by evolution. A mutation that results in cooperative binding between a pair of transcription factors has two effects on the fitness of a transcriptional circuit. For a target that is regulated by both transcription factors, we assume that cooperative binding mitigates the effects of deleterious mutations at transcription factor binding sites [7-9]. This results in a reduced fitness penalty for a mutation at the *β*(*K*_1_ + *K*_2_) shared targets, so that (1−*s*) is replaced by (1−*hs*) for some constant 0≤*h*≤1. Nonetheless, there are also (1−*β*)(*K*_1_ + *K*_2_) targets that are regulated by only one or the other of the transcription factors. We assume that the cooperative binding of the transcription factors causes pleiotropic mis-regulation at these targets (since the other transcription factor, which does not have a binding site at such sites, now binds to the first transcription factor through a physical interaction). This results in a fitness penalty *t* at each of the (1−*β*)(*K*_1_ + *K*_2_) targets that are not co-regulated. Fitness is again assumed to be multiplicative, so that the cost of pleiotropy associated with cooperative binding is ${\left(1-t\right)}^{\left(1-\beta )(K_{1}+K_{2}\right)}$.

Provided *u*_*l*_,*u*_*g*_ ≪ 1, genes that are co-regulated and genes that are not co-regulated have equilibrium distributions described by independent binomial distributions with means *a*_*hs*_and *a*_*s*_respectively, which are approximated by Equation 2 (substituting *hs* for *s* appropriately, see Methods). We can now specify the conditions for the invasion of cooperative gene regulation. A mutation resulting in cooperative binding between a pair of factors will be favoured if the expected fitness of the mutant is greater than the equilibrium mean fitness. Using the expressions for mean fitness given above, this occurs when ${\left(1-a_{s}s\right)}^{\beta (K_{1}+K_{2})}<{\left(1-t\right)}^{\left(1-\beta )(K_{1}+K_{2}\right)}{\left(1-a_{s}\mathrm{hs}\right)}^{\beta (K_{1}+K_{2})}$. Assuming *t*,*s* ≪ 1, this expression can be simplified to give $\beta >\frac{t}{t+sa_{s}(1-h)}$. This means that, when the fraction of binding sites at shared targets, *β*, is greater than a threshold depending on *s*, *h*, *t* and *a*_*s*_, a mutation that results in cooperative binding can invade a population at equilibrium.

Similarly, a mutation that results in the loss of cooperative binding in a population where it is present will be favoured when ${\left(1-a_{\mathrm{hs}}s\right)}^{\beta (K_{1}+K_{2})}<{\left(1-t\right)}^{\left(1-\beta )(K_{1}+K_{2}\right)}{\left(1-a_{\mathrm{hs}}\mathrm{hs}\right)}^{\beta (K_{1}+K_{2})}$. Again assuming *t*,*s* ≪ 1, this expression can be simplified to give $\beta <\frac{t}{t+sa_{\mathrm{hs}}(1-h)}$ so that, when the fraction of binding sites at shared targets, *β*, is less than a threshold depending on *s*, *h*, *t* and *a*_*hs*_, a mutation that results in loss of cooperative binding can invade a population at equilibrium.

Since the first expression in Equation 2 is monotonically decreasing in *s*, and the second expression is independent of *s*, it is always true that *a*_*hs*_≤*a*_*s*_, i.e populations that have cooperative binding accumulate more deleterious mutations, that result in weaker transcription factor binding sites, than populations that lack it. As a result there is a range of *β* for which *both* a population that lacks cooperative binding, *and* a population that has cooperative binding are not invadable by mutations that gain or remove cooperative binding respectively. In this range, the evolutionary dynamics of the system are bi-stable. In this range, we expect to find some genes that are regulated by pairs of transcription factors that act cooperatively and some that don’t.

Using the expression for *a*_*s*_ given in Equation 2, and recalling that *a*_0_ = *u*_*l*_/(*u*_*l*_+*u*_*g*_) is the neutral equilibrium in a system dominated by drift, the threshold value of *β* above which selection favours a mutation causing cooperative binding in a population that lacks it, is given by

(3) $$\beta >\left\{\begin{array}{c} \frac{2\mathrm{Nt}}{2\mathrm{Nt}+a_{0}(1-h)}\;\;\text{if}\;2\mathrm{Ns}>1\\ \frac{t}{t+sa_{0}(1-h)}\;\;\;\text{otherwise} \end{array}\right).$$

Similarly, the threshold value of *β* below which selection favours a mutation resulting in loss of cooperative binding in a population that has it, is given by

(4) $$\beta <\left\{\begin{array}{c} \frac{2\mathrm{Nth}}{2\mathrm{Nth}+a_{0}(1-h)}\;\;\text{if}\;2\mathrm{Nhs}>1\\ \frac{t}{t+sa_{0}(1-h)}\;\;\;\;\text{otherwise} \end{array}\right).$$

**Figure 2:**
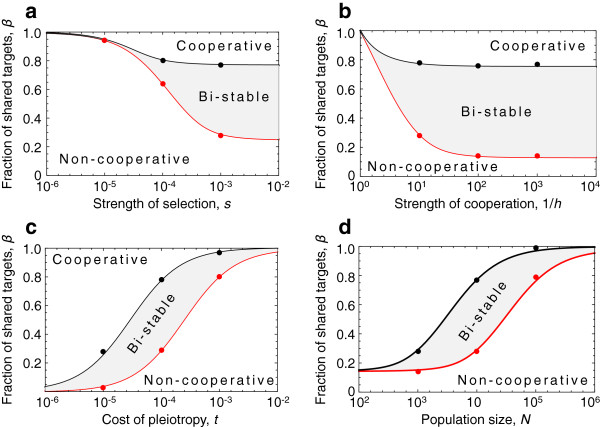
**Evolutionary parameters that permit cooperative regulation.** Evolutionary parameters that permit the evolution of gene regulation by cooperative transcription factors. Threshold number of shared targets for gain (black) and loss (red) of cooperative binding to be advantageous in a population at equilibrium under stabilising selection. The black line shows the value of *β*above which a new mutation that results in cooperative binding will invade in a population that lacks cooperative binding. The red line shows the value of *β*below which a mutation resulting in loss of cooperative binding will invade, in a population that has cooperative binding. For values of *β*that lie in the gray region, the dynamics are bistable: a population with cooperative binding will preserve it, and one without binding will not gain binding. The threshold fraction of shared targets varies with (top left) strength of selection, *s*, (top right) strength of cooperativity in reducing the effects of deleterious mutations 1/*h*, (bottom left) the cost of pleiotropy *t* and (bottom right) the population size, *N*. Lines show our analytic equations (Equations 2 and 3), and points show the results of 10^5^replicate Monte-Carlo simulations. Parameter values (unless stated otherwise) are *u*_*l*_=2×10^−7^, *u*_*g*_=10^−7^, *K*_1_ + *K*_2_=100, *s*=10^−3^, *h*=10^−1^, *t*=10^−4^and *N*=10^4^.

These equations allow us to make a number of observations about the evolution of cooperative gene regulation (Figure 2, and see Methods). Beginning with Equation 3 for a population lacking cooperative binding, we see that when *N* and/or *s* is large, so that 2*Ns* > 1, the threshold number of shared targets *β*above which cooperative binding becomes advantageous is independent of the strength of selection *s* (Figure 2a). However the threshold decreases as the mutation-buffering effect of cooperative binding increases (i.e. as *h* decreases, Figure 2b). As population size *N* increases, selection becomes more efficient and the threshold value of *β* increases (Figure 2c). Finally, the threshold also increases with the cost of pleiotropy *t* (Figure 2d). In contrast, when *N* and/or *s* is small, so that 2*Ns* < 1, drift dominates and the threshold number of shared targets *β*is independent of population size *N* (Figure 2c). However the threshold decreases with the strength of selection *s* (Figure 2a), because when drift dominates the number of deleterious mutations is at the neutral equilibrium, and increasing *s* increases the impact of each mutation on overall fitness.

Similarly, from Equation 4 for a population with cooperative binding, we see that when *N* and/or *hs* is large, so that 2*Nhs* > 1, the threshold number of shared targets *β*below which cooperative binding becomes disadvantageous is independent of the strength of selection *s* (Figure 2a). As before, the threshold decreases as the mutation buffering effect of cooperative binding increases (i.e. as *h* decreases, Figure 2b) and the threshold increases with population size *N* (Figure 2c), and the cost of pleiotropy *t* (Figure 2d). In contrast, when *N* and/or *hs* is small, so that 2*Nhs* < 1, drift dominates and the threshold number of shared targets *β*is independent of population size *N* (Figure 2c), but decreases with the strength of selection *s* (Figure 2a). The size of the bistable region is largest when *s* is large and *h* is small, and for intermediate values of *N* and *t*, as shown in Figure 2. As this analysis demonstrates, there is a broad range of possible evolutionary outcomes and, crucially, cooperative binding can evolve under a wide range of circumstances despite the deleterious pleiotropic effects associated with physical interactions among transcription factors.

### Adaptation of transcriptional circuits under positive selection

**Figure 3:**
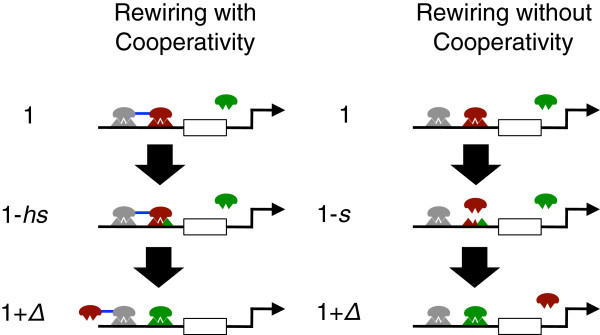
**A schematic cartoon of rewiring.** A schematic cartoon of rewiring with (left) and without (right) cooperative binding. Selection favours a change in the regulation of target genes from the red TF to the green TF. Rewiring requires an initially deleterious mutation at the red binding site before a green binding site can be acquired. The fitness of the different states is shown on the left hand side for each case. The reduced fitness of the intermediate state is less when cooperative binding is present than when it is absent.

When cooperative binding is present, under stabilising selection, transcription factor binding sites at co-regulated genes are better able to tolerate mutations (i.e *a*_*hs *_>*a*_*s*_). Under positive selection for a novel expression phenotype, this may speed adaptation, since greater mutational robustness generates greater genetic diversity and can help speed adaptation (Figure 3a) [22]. This may occur, for example, when adaptation involves change in the transcription factor that regulates a target gene [7-9,11], through turnover of transcription factor binding sites [23-25]. We use our model to quantify the extent to which cooperative binding among transcription factors accelerates the adaptive rewiring of transcriptional circuits under positive selection.

We study adaptive change that involves replacement of an existing transcription factor by a new one that confers higher fitness. We assume that the target gene must first suffer an initially deleterious mutation at its existing binding site before a newly adaptive binding site can be acquired (Figure 3) [8,9,11]. The newly adaptive binding site is produced from binding sites that have already mutated at a rate *u*_*r*_. The expected waiting time for such a gene to produce a newly adaptive binding site therefore depends on the number of binding sites in the population that harbor a deleterious mutation, which is proportional to *a*_*s*_ when cooperativity is absent and *a*_*hs*_ when it is present. Since *a*_*hs *_>*a*_*s*_, this number is greater when cooperative binding is present than when it is absent.

**Figure 4:**
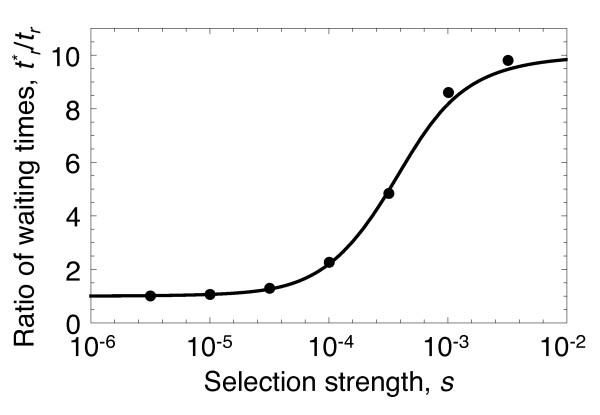
**Cooperative binding accelerates adaptation.** Cooperative binding accelerates adaptation under positive selection. The ratio of waiting times before the arrival of novel adaptive binding sites for populations without ( $t_{r}^{*}$) and with (*t*_*r*_) cooperative binding. Provided *Ns* > 1, cooperative binding reduces the adaptation time up to 10-fold, compared to populations that lack cooperative binding. The line shows our analytic expression, and points show the result of 10^5^replicate Monte-Carlo simulations. Parameter values *u*_*l*_=2×10^−7^, *u*_*g*_=10^−7^, *K*_1_ + *K*_2_=100, *h*=10^−1^, *t*=10^−4^, *N*=10^4^, *u*_*r*_=10^−7^.

The ratio of waiting times before a newly adaptive binding site arises, $t_{r}^{*}$/*t*_*r*_ (for populations without, $t_{r}^{*}$, or with, *t*_*r*_, cooperative binding), quantifies the degree to which cooperative binding of transcription factors accelerates adaptation under positive selection. This ratio is given by *a*_*hs*_/*a*_*s*_ (Figure 4, see Methods). As Figure 4 shows, provided *Ns* > 1 (i.e. provided deleterious mutations at binding sites are not nearly neutral), rewiring of transcriptional circuits is significantly accelerated by cooperative binding among transcription factors. Thus, a population that has cooperative binding among transcription factors under stabilizing selection, can also experience an accelerated rate of adaptation.

### Cooperative binding and the fraction of shared targets in yeast

**Figure 5:**
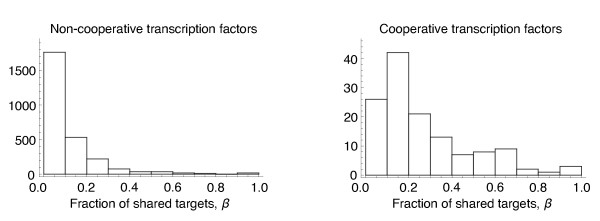
**Number of shared targets.** Fraction of targets that are shared between pairs transcription factors in *S. cerevisiae*[26-28]. (left) The fraction of targets that are shared among paris of transcription factors that lack cooperative binding and (right) the fraction of targets that are shared among transcription factors that bind cooperatively. The fraction of targets that are shared is larger among cooperative factors (*p* < 2×10^−16^, Wilcoxon test).

Our model predicts that, under stabilising selection, cooperative binding will be favoured when the fraction of targets shared by a pair of transcription factors exceeds a certain threshold. In order to test this prediction, and to get some idea of the degree of overlap that is required for cooperative binding to arise in natural systems, we inspected pairs of transcription factors in *Saccharomyces cerevisiae*. A total of 186 pairs are reported as participating in cooperative binding [26], based on a combination of ChIP-chip data, transcription factor knockout data, and direct experimental evidence. Using the set of genes regulated through a transcription factor binding site for a total of 204 yeast transcription factors [27,28], we determined the fraction of overlapping targets, *β*, for all pairs of transcription factors (Figure 5). It is important to note that, typically, studies that systematically look for cooperative gene interactions take into account the number of targets shared by a gene pair. Therefore, to minimise the risk of circularity in our analysis, we have used separate datasets to determine cooperative gene interactions, and to determine regulatory targets. The mean fraction of overlapping targets for genes identified as participating in cooperative binding was 10-fold greater (0.21) than the mean fraction of overlapping targets at genes that do not bind cooperatively (0.02) which is highly statistically significant (*p* < 2×10^−16^, Wilcoxon test). This supports the prediction of our population-genetic analysis, and it suggests that a sizeable overlap in targets is required before cooperative binding becomes advantageous.

### Cooperative binding in the yeast sex determination network

The ability of cooperative transcription factors to facilitate adaptation also has empirical support, from observations in the sex determination networks of different yeast species [7-9]. The acquisition of a protein-protein interaction between the mating factor MAT*α*2 and Mcm1 was able to buffer the deleterious effects of mutations that strengthened Mcm1 binding sites [7]. Prior to the emergence of a protein-protein interaction, sex determining genes were activated only in the presence of Mcm1 and MATa2 together [7]. The buffering effects of the protein-protein interaction allowed Mcm1 binding sites to acquire strengthening mutations such that sex determining genes became activated by Mcm1 alone. As a result, MATa2 became redundant and was lost [7]. The result was a significant upstream reorganization of the yeast sex determination network without the need for any parallel changes to the downstream output of the network. Similar patterns, in which acquisition of cooperative binding between transcription factors is followed by changes to the regulation of their shared targets, are observed across the yeast transcriptome [8], and support the prediction of our analysis of positive selection on transcriptional networks.

## Conclusions

We have shown that cooperative binding between a pair of transcription factors is favoured under stabilising selection, provided the overlap between their targets is sufficiently large. The threshold fraction of shared targets depends upon the strength of selection on binding sites, the cost of pleiotropy associated with protein-protein interactions, and the rates of mutations. It also depends on the population size. Just as in models that consider the evolution of redundancy [20,29], we find that greater redundancy (i.e. cooperative regulation) is more strongly favoured in smaller populations; and that for intermediate population sizes the evolutionary dynamics are bistable, such that cooperative binding is maintained if it is already present, but cannot evolve if it is absent. Finally, we found that cooperative binding facilitates the rewiring of transcriptional circuits under positive selection.

This study shows that, even when the deleterious effects of pleiotropy are taken into account, mutations that change transcription factor function can play an important role in the evolution of gene expression. Taking account of mutations both to regulatory binding sites and to the transcription factors themselves reveals a rich set of evolutionary dynamics that helps explain how complex transcriptional networks can rapidly rewire large sets of genes in order to adapt to new environments.

## Methods

### Equilibrium distribution

To find the equilibrium relative abundances of the hamming classes *x*_*i*_that give the solution to Equation 1, we follow [20,21] and look for a solution of the form $x_{i}=\left(\begin{array}{c} K_{1}+K_{2}\\ \;\;\;i \end{array}\;\right)a_{s}^{i}{\left(1-a_{s}\right)}^{K_{1}+K_{2}-i}$. Given this assumed form, the mean fitness of the population at equilibrium is $\bar{\omega }=\underset{i}{\sum }{\left(1-s\right)}^{i}x_{i}$. Since ∑_*i*_*x*_*i*_=1 it is easy to show that $\underset{i}{\sum }\left(\begin{array}{c} K_{1}+K_{2}\\ \;\;\;i \end{array}\;\right)p^{i}={\left(1+p\right)}^{n}$. Taking *p*=*a*(1−*s*)/(1−*a*), this gives a mean fitness of $\bar{\omega }={\left(1-a_{s}s\right)}^{\left(K_{1}+K_{2}\right)}$, which is the form given in the main text.

To compute *a*_*s*_ we follow [20] and write down the generating function *π*_*V*_(*z*) of a random variable *V * defined by the Hamming class after mutation of an individual chosen from the population according to its relative fitness, where *z* is a formal variable. The function *π*_*V*_(*z*) may be thought of as a probability generating function, where the probability distribution associated with it gives the distribution of Hamming classes in the population at equilibrium [20]. In the case of non-identical forward and backward mutation rates, *u*_*l*_ and *u*_*g*_, this is given by $\pi_{V}(z)=\underset{i}{\sum }w_{i}x_{i}{\left(u_{l}+(1-u_{l})z\right)}^{i}{\left((1-u_{g})+u_{g}z\right)}^{K_{1}+K_{2}-i}$. Following [20], we analyse the eigensystem problem associated with the population dynamics to determine the equilibrium distribution of Hamming classes (i.e the distribution of genotypes in the population under mutation selection balance). In equilibrium we have *π*_*V*_(*z*)=*λ*∑_*i*_*x*_*i*_*z*^*i*^, where *λ*is the eigenvalue associated with the system. Using our assumed form of *x*_*i*_ results in the infinite population equilibrium distribution:

(5) $$\;a_{s}=\frac{1}{2}\;\left[\;1\;+\;\frac{u_{l}+u_{g}}{s}-u_{g}-\sqrt{{\left(1\;+\;\frac{u_{l}+u_{g}}{s}-u_{g}\right)}^{2}\;\;\;-\;\frac{4u_{l}}{s}}\right]$$

When cooperative binding is present a subset *β*(*K*_1_ + *K*_2_)=*K*_*hs*_of the target genes have selective coefficient *hs* and the remaining (1−*β*)(*K*_1_ + *K*_2_)=*K*_*s*_have selective coefficient *s*. The hamming class of an individual now has two indices *i* and *j* such that *x*_*ij*_ refers to an individual with *i* mutations at shared targets and *j* mutations at unshared targets. In this case we look for solutions of the form $x_{\mathrm{ij}}=\left(\begin{array}{c} K_{\mathrm{hs}}\\ \;\;i \end{array}\;\right)a_{\mathrm{hs}}^{i}{\left(1-a_{\mathrm{hs}}\right)}^{K_{\mathrm{hs}}-i}\left(\begin{array}{c} K_{s}\\ \;\;j \end{array}\;\right)a_{s}^{j}{\left(1-a_{s}\right)}^{K_{s}-j}$. The generating function of *V * is now given by

$$\begin{array}{l} \;\pi_{V}(z_{\mathrm{hs}},z_{s})=\underset{i}{\sum }\underset{j}{\sum }w_{\mathrm{ij}}x_{\mathrm{ij}}{\left(u_{l}+(1-u_{l})z_{\mathrm{hs}}\right)}^{i}\\ \;\times {\left((1-u_{g})+u_{g}z_{\mathrm{hs}}\right)}^{K_{\mathrm{hs}}-i}{\left(u_{l}+(1-u_{l})z_{s}\right)}^{j}\\ \;\times {\left((1-u_{g})+u_{g}z_{s}\right)}^{K_{s}-j} \end{array}$$

and at equilibrium $\pi_{V}(z_{\mathrm{hs}},z_{s})\;=\;\lambda \;\underset{i}{\sum }\;\underset{j}{\sum }\;x_{\mathrm{ij}}\;z_{\mathrm{hs}}^{i}\;z_{s}^{j}$. Because we are assuming that *w*_*ij*_is just the product of the two independent fitness landscapes associated with the different selective coefficients, i.e *w*_*ij*_=(1−*hs*)^*i*^(1−*s*)^*j*^ using our assumed form of *x*_*ij*_ results in values of *a*_*s*_and *a*_*hs*_ as given by Equation 5 for the independent distributions with the appropriate selective coefficients.

The finite *N* approximation of Equation 5, can be obtained from the moment equations of Woodcock and Higgs [21], assuming *u*_*l*_*u*_*g*_*sN*^−1^ ≪ 1. This gives

(6) $$\;\begin{array}{l} a_{s}=\frac{1}{2}\left[1+\frac{1+2(u_{l}+u_{g})}{2\mathrm{Ns}}\right)\\ \;-\left(\sqrt{{\left(\;1\;+\;\frac{1+2(u_{l}+u_{g})}{2\mathrm{Ns}}\;\right)}^{2}\;\;\;-\;\frac{4u_{l}}{u_{l}\;+\;u_{g}}\frac{1\;+\;2(u_{l}+u_{g})}{2\mathrm{Ns}}}\right]\;. \end{array}$$

Assuming *u*_*l*_,*u*_*g*_ ≪ *s*, we obtain the Taylor expansion of *a*_*s*_ to first order, in terms of 1/(2*Ns*) (which is relevant when 2*Ns* > 1) and in terms of *2Ns* (relevant when 2*Ns* < 1) to obtain Equation 2.

Using the above distributions, the equilibrium mean fitness *w*_*ind*_, in the absence of cooperative binding is $w_{\mathrm{ind}}={\left(1-a_{s}s\right)}^{K_{1}+K_{2}}$, and in the presence of cooperative binding, *w*_*coop*_is $w_{\mathrm{coop}}={\left(1-t\right)}^{\left(1-\beta )(K_{1}+K_{2}\right)}{\left(1-a_{s}s\right)}^{\left(1-\beta )(K_{1}+K_{2}\right)}{\left(1-a_{\mathrm{hs}}\mathrm{hs}\right)}^{\beta (K_{1}+K_{2})}$ which can be Taylor expanded to first order and, combined with Equation 2, give Equations 3 and 4. We can also find the conditions for the equilibrium mean fitness of a population with cooeprative binding to be greater than that for a population that lacks it, i.e *w*_*coop*_ >*w*_*ind*_. When *t*,*s* ≪ 1 this can be expressed as

(7) $$\beta >\left\{\;,\begin{array}{c} \frac{2\mathrm{Nt}}{2\mathrm{Nt}+a_{0}(1-2\mathrm{Nhs})}\;\;\text{if}\;2\mathrm{Ns}>1\;\text{and}\;2\mathrm{Nhs}<1\\ 1\;\;\;\;\;\;\text{otherwise} \end{array}\right).$$

According to this inequality, cooperative binding is advantageous only when the fraction of targets shared by the pair of transcription factors is greater than a threshold. Since by definition *β*≤1, Equation 7 says that cooperative binding can only increase population mean fitness when 2*Nhs* < 1 and 2*Ns* > 1, i.e if the benefit of cooperativity, *h*, is sufficient to make mutations at transcription factor binding sites that are deleterious in the absence of cooperativity nearly neutral when it is present.

### Rewiring time

For a given binding site the waiting time *T* for the arrival of the first adaptively rewired mutant to arise is given by the distribution

$$P\{T>t\}=E\;\left[e^{-\mathrm{\zeta Yt})}\right]\;,$$

where *t* is time, *ζ*is the rate at which the rewiring mutations occur and *Y * is fraction of the population at equilibrium that is able to undergo rewiring mutations. We assume rewiring mutations can occur only following an initially deleterious mutation. In the absence of cooperativity, the fraction of the population with a mutation at a given site is *a*_*s*_, therefore *Y*∝*Na*_*s*_, and we are able to write

$$P\{T>t\}=\;E\left[e^{-u_{r}Na_{s^{t}}}\right]\;,$$

where *u*_*r*_ gives the rate at which rewiring mutations occur at sites that have already undergone an initially deleterious mutation. The excepted waiting time for a single gene is thus

$$T_{s}=\frac{1}{u_{r}Na_{s}}$$

If the gene to be rewired is coregulated by a pair of transcription factors that bind cooperatively, we similarly have

$$T_{\mathrm{hs}}=\frac{1}{u_{r}Na_{\mathrm{hs}}}$$

and the ratio of waiting times *T*_*s*_/*T*_*hs*_ is therefore simply *a*_*hs*_/*a*_*s*_. Finally, if *k* genes must be rewired before adaptation occurs, the waiting time for the first event is *T*/*k*(where *T* is the waiting time for 1 gene to rewire), the waiting time for the second event is *T*/(*k*−1) and so on. Therefore the total expected waiting time, *T*_*s*_(*k*) is

$$T_{s}(k)=\frac{1}{u_{r}Na_{s}}\overset{k}{\underset{i=1}{\sum }}\frac{1}{i}$$

Therefore the ratio of expected waiting times with and without cooperative binding is independent of the number of genes to be rewired, and depends only on the ratio *a*_*hs*_/*a*_*s*_.

### Variation in selection strength across sites

Up to this point we have assumed that the selective coefficients, *s* and *h*, are constant across binding sites. However it is obviously possible that these parameters may vary between binding sites. Such a generalization of our model represents a significant complication, and a full treatment is outside the scope of this paper. However, it can be analyzed in the simple case that the coefficients associated with each binding site *i* satisfy *s*_*i*_ ≪ 1, such that the fitness landscape is approximately additive.

We assume that there are a finite set of selective coefficients, *s*^*α*^ and *h*^*γ*^, where the super-scripts *α*and *γ*index the different sets, and that the number of binding sites with a given coefficient *s*^*α*^ or *h*^*γ*^are distributed according to some function *F*(*s*) and *G*(*h*). We also assume that the coefficients *s* and *h* are distributed independently of one another. Each binding site *i* has a value *s*_*i*_ associated with it, drawn according to *F*(*s*). In the quasi-species regime the probability that binding site *i* has a mutation is simply given by $a_{s_{i}}$, as given by Equation 5. In this case the distribution of hamming classes, rather than being binomial, is poisson binomial, paramaterized by $a_{s_{i}}$. Similarly, when cooperative binding is present, the distribution is poisson binomial with the modification that shared targets have a mutation with probability $a_{s_{i}h_{i}}$ where *h*_*i*_ is drawn independently from the distribution *G*(*h*). The system is easiest to analyse if we separate binding sites into sub-classes, *α*, of binding sites with the same selective coefficients, where the size of each sub-class, *n*_*α*_ is given by *n*_*α*_=*F*(*s*^*α*^)(*K*_1_ + *K*_2_). The number of mutations in each subclass is then given by a binomial distribution.

When the fitness landscape is close to additive, the method of [19] can be applied independently to each sub-class to determine the expected number of mutations in the sub-class. This is only true in an additive fitness landscape, or in a multiplicative fitness landscape in which cross terms between subclasses are sufficiently small that they can be neglected. When this condition holds, the value of $a_{s^{\alpha }}$ associated with each sub-class is given by Equation 6.

The expected number of mutations in each subclass is simply $a_{s^{\alpha }}n_{s^{\alpha }}$ and the expected fitness of each sub-class is ${\left(1-a_{s^{\alpha }}s^{\alpha }\right)}^{n_{\alpha }}$. The expected mean fitness of the population is then $\bar{\omega }=\underset{\alpha }{\prod }{\left(1-a_{s^{\alpha }}s^{\alpha }\right)}^{n_{\alpha }}$. Using our almost additive assumption, this can be approximated by $\bar{\omega }\approx 1-(K_{1}+K_{2})\underset{\alpha }{\sum }F(s^{\alpha })a_{s^{\alpha }}s^{\alpha }$. This is the fitness of the population when cooperative binding is absent. Similarly, when cooperative binding is present we have,

$$\begin{array}{l} \;w_{\mathrm{coop}}\approx 1-(1-\beta )(K_{1}+K_{2})t-(1-\beta )(K_{1}+K_{2})\\ \;\times \underset{\alpha }{\sum }F(s^{\alpha })a_{s^{\alpha }}s^{\alpha }+\beta (K_{1}+K_{2})\\ \;\times \underset{\alpha }{\sum }\underset{\gamma }{\sum }F(s^{\alpha })G(h^{\gamma })a_{h^{\gamma }s^{\alpha }}h^{\gamma }s^{\alpha } \end{array}$$

From these the invasion probabilities and threshold values of *β*can be calculated in the same way as in the case of constant *s* and *h* above. The only difference in the case with variable selective coefficients is that the invisibility criteria for a mutation resulting in gain or loss of cooperative biding dependants on the average of *a*_*s*_*s* across the distribution *F*(*s*), and on the average *a*_*hs*_*hs*across the joint distribution *F*(*s*)*G*(*h*). Investigating different forms of the functions *F*(*s*) and *G*(*h*) represents an interesting avenue for further work.

## Authors’ contributions

AJS, AP, RMS, and JBP designed the research. AJS and RMS performed the analysis. AJS, AP, and JBP wrote the paper. All authors read and approved the final manuscript.
